# *Ocimum sanctum* Linn. ethanolic extract inhibits angiogenesis in human lung adenocarcinoma (a549) cells

**DOI:** 10.14202/vetworld.2020.2028-2032

**Published:** 2020-09-30

**Authors:** Hevi Wihadmadyatami, Puspa Hening, Ulayatul Kustiati, Dwi Liliek Kusindarta, Teguh Triyono, Supriatno Supriatno

**Affiliations:** 1 Department of Anatomy, Faculty of Veterinary Medicine, Universitas Gadjah Mada, Yogyakarta, Indonesia; 2 Integrated Laboratory for Research and Testing, Universitas Gadjah Mada, Yogyakarta, Indonesia; 3 Department of Clinical Pathology, Faculty of Medicine, Universitas Gadjah Mada, Yogyakarta, Indonesia; 4 Department of Oral Medicine, Faculty of Dentistry, Universitas Gadjah Mada, Yogyakarta Indonesia

**Keywords:** A549 cells, angiogenesis, integrin αvβ3, matrix metalloproteinase, *Ocimum sanctum* ethanolic extract

## Abstract

**Background and Aim:**

*Ocimum sanctum* (OS) is a herbal plant, which is easy to find and is widely used as an alternative medication. The previous studies have shown that several species of OS extract have therapeutic properties, and in some cases, antitumor properties. Furthermore, several data have shown the antiproliferative effects of OS extract in cases of breast cancer, human fibrosarcoma, and oral cancer. Lung adenocarcinoma is a major cause of male cancer worldwide; however, the effect of OS (of Indonesian origin) on the metastasis of human alveolar pulmonary adenocarcinoma A549 cells remains unclear. This study aimed to analyze the antiangiogenic effects of OS ethanolic extract in A549 lung adenocarcinoma cells.

**Materials and Methods:**

An angiogenesis assay was performed by seeding A549 cells on extracellular matrix solution and observing tube formation using an inverted microscope. Enzyme-linked immunosorbent assay for αvβ3, matrix metalloproteinase (MMP)-2, and MMP-9 was performed by analyzing the cell lysate after a given treatment.

**Results:**

OS ethanolic extract significantly inhibited tube formation of A549 cells and suppressed the expression of integrin αvβ3, MMP-2, and MMP-9.

**Conclusion:**

Our findings indicate that OS ethanolic extract disrupts angiogenesis of A549 cells, which may result from the disruption of cell migration and proliferation as a consequence of downregulation of αvβ3, MMP-2, and MMP-9. Taken together, OS ethanolic extract may represent a good therapeutic candidate for the treatment of metastasis in lung adenocarcinoma. Further studies are warranted to further establish the efficacy of OS in the treatment of lung adenocarcinoma.

## Introduction

Human lung adenocarcinoma is one of the most common types of lung cancer and has high metastatic potential. Lung cancer remains the leading cause of cancer mortality in men and women in the U.S. and worldwide. Approximately 90% of lung cancer cases are caused by smoking and the use of tobacco products, and almost 60% of patients with lung cancer have late-stage disease at the time of diagnosis. Lung cancer is primarily treated by surgery, radiation, and chemotherapy; however, these treatments are often unsatisfactory in patients with advanced metastasis [1,2].

Tumor angiogenesis pathways have been identified as important therapeutic targets in many cancers. Angiogenesis is defined as the formation of new capillaries from pre-existing vasculature. Angiogenesis has important physiological roles in wound healing, embryogenesis, and organogenesis [3,4]; however, in pathologic conditions, such as cancer, tumor growth, and metastasis depend on angiogenesis, which is triggered by chemical signals from tumor cells. Without blood vessels, tumors cannot grow beyond a critical size or metastasize to another organ [5]. To grow or locally metastasize, tumor tissues also require oxygen and nutrients, which are provided by blood vessels. In growing cancers, endothelial cells are vigorously active as a result of the release of proteins including epidermal growth factor, estrogen, basic and acidic fibroblast growth factors, interleukin-8, tumor necrosis factor−α, and vascular endothelial growth factor (VEGF), which can activate endothelial cell growth and motility when the production of antiangiogenic factors is reduced [6,7].

During angiogenesis, αv integrins are overexpressed on the endothelial cell surface to facilitate the growth than the survival of new vessels [8]. Integrins are important in the attachment of cells to each other as well as to their surrounding extracellular matrix (ECM) [9]. Integrins are heterodimeric transmembrane receptors consisting of 18 alpha and 8 beta subunits [10]. Integrins mediate signals for the control of diverse cellular functions, including survival, proliferation, differentiation, adhesion, and migration [11-13]. The αvβ3 integrin is most abundantly expressed on angiogenic endothelial cells during remodeling and in pathological tissues, including cancer. Several medications target αvβ3 integrin by blocking its activities using monoclonal antibodies or cyclic Arginine-Glycine-Aspartate (cRGD) peptide. Inhibition of αvβ3 integrin activities may lead to cell detachment and inhibit tube formation in angiogenesis [14].

*Ocimum sanctum* (OS) Linn. ethanolic extract is a herbal extract that originates from a plant native to Indonesia. OS has many benefits as a traditional medicine, and the previous studies have shown that several species of OS have antiproliferative and anticancer effects on oral cancer cell line and MDA-MB-435 breast cancer cell line and reduce invasion and matrix metalloproteinase (MMP) activity in head-and-neck cancer cell lines [15,16]. In addition, OS has been reported to have neuroprotective, neuroregenerative, antioxidant, and anti-inflammatory effects.

Since OS has diverse effects in many pathological conditions, we sought to investigate the effect of OS ethanolic extract on angiogenesis in human lung adenocarcinoma (A549) cells.

## Materials and Methods

### Ethical approval

No ethical approval was required for the study.

### Study period and location

The study was conducted in the Integrated Laboratory for Research and Testing, Universitas Gadjah Mada, from March 2019 to February 2020.

### OS leaf extraction

OS leaves were acquired from the Center for Research and Development of Medicinal Plants and Traditional Medicines, Ministry of Health in Tawangmangu, Central Java, Indonesia. The OS type was identified in the Plant Systematics Laboratory, Faculty of Biology, Universitas Gadjah Mada. The specimen vouchers were saved in the Center for Agro Technology Innovation, Universitas Gadjah Mada (ID number: UGM-00430). Crude extracts and ethanolic extracts of OS were prepared as previously described [17]. The ethanolic extracts were then diluted with phosphate buffer saline (PBS) pH 7.4 to prepare the required concentrations (50 μg/mL, 70 μg/mL, 100 μg/mL, and 200 μg/mL) (Gibco, California, USA).

### Cell line maintenance

The A549 cell line was a generous gift from Prof. Srikanth Karnati (Wurzburg, Germany). A549 cells were cultured in Dulbecco’s Modified Eagle Medium supplemented with 10% fetal bovine serum (Gibco, California, USA) at 37°C and 5% CO_2_. Cells were divided into eight groups: Normal cells in non-Matrigel-coated wells as negative control (non-treated cells: NT); normal cells in Matrigel-coated wells treated with VEGF (Abcam, Cambridge, USA) as the positive control of angiogenesis; cells treated in Matrigel-coated wells in the presence of cRGD (Bachem, Bubendorf, Switzerland) as negative control; and cells treated with 50 μg/mL, 70, μg/mL, 100 μg/mL, and 200 μg/mL OS ethanolic extract.

### Angiogenesis assay

The angiogenesis assay was performed using the Angiogenesis Assay Kit (Abcam, Cambridge, USA) and follows: 50 μL of cold ECM solution was added to cold 96-well plates (Thermo Fisher Scientific, New York, USA) and incubated for 1 h in a CO_2_ incubator. 10^5^ cells were seeded in each Matrigel-coated well, and treatments (non-treated [NT], OS ethanolic extract 50 μg/mL, 70 μg/mL, 100 μg/mL, and 200 μg/mL) were added after 30 min of incubation. The plates were incubated for 18 h and analyzed using an inverted microscope with 10× (Zeiss, Jena, Germany). For quantification, data were imported as TIFF files into ImageJ (http://imagej.nih.gov/ij/) using the stage micrometer as a calibrator.

### Preparation of cell lysates

A549 cells (5 × 10^5^) were seeded in each well of a 6-well plate, then incubated for 1 h. The treatments outlined above were added to each well (one treatment per well) and incubated for 24 h. The media were then aspirated and the plates were washed using PBS (Gibco, California, USA). Following washing, 600 μL RIPA buffer (Gibco, California, USA) was added, and the plate was shaken and rocked for 15 min. Cell scrapers were used to detach the cells from the bottom of the plate, and the lysates were transferred to 1.5 mL microtubes. The plates were washed using 300 μL RIPA buffer and then combined with the first lysate. The lysates were centrifuged at 10.000× *g* for 10 min at 4°C, and the supernatants were transferred to 1.5 mL microtubes.

### Enzyme-linked immunosorbent assay (ELISA)

ELISAs were performed using human ITG αvβ3, human MMP-2, and human MMP-9 ELISA kits (Fine Test, Wuhan, China). The plates were washed twice before adding standard, sample, and control (zero) wells. One hundred microliters standard or sample was added to each well and incubated for 90 min at 37°C. The wells were aspirated, and the plates were washed twice. One hundred microliters biotin-labeled αvβ3 antibody working solution was added to each well and incubated for 60 min at 37°C. The wells were aspirated, and the plates were washed 3 times. One hundred microliters HRP-streptavidin conjugate, streptavidin-biotin complex working solution was added to each well and incubated for 30 min at 37°C. The wells were aspirated, and the plates were washed 5 times. Ninety microliters tetramethylbenzidine substrate was added and incubated for 15-30 min at 37°C. Fifty microliters stop solution was added, and the plate was read immediately at 450 nm. Backgrounds (negative controls) without the sample were run for cell lysates in parallel to correct for nonspecific binding. Cutoff values were determined as the mean signal of negative control (n=10) plus three standard deviations.

### Statistical analysis

Multiple statistical comparisons were made using one-way analysis of variance followed by Tukey’s *post hoc* test as appropriate. p<0.05 was assumed to represent statistical significance. Statistical analysis was performed using GraphPad Prism 6 (La Jolla, CA, USA).

## Results

### OS Extract inhibits angiogenesis of A549 Cells

**Figure-1:**
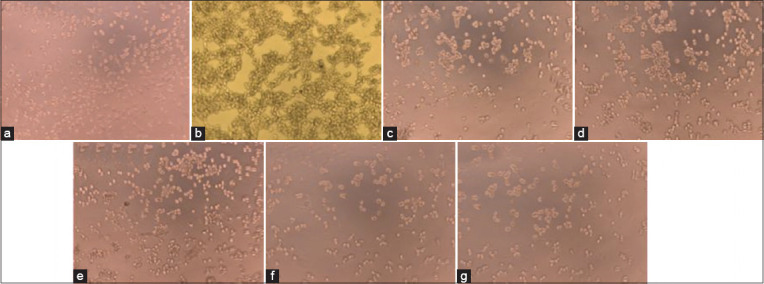
*Ocimum sanctum* extract inhibits angiogenesis of A549 lung cancer cells. 1×10^5^ cells were seeded in each well coated with Matrigel, and the background control wells without Matrigel. Each treatment was added following 30 min of incubation, and the plates were incubated for 18 h at 37°C, 5% CO_2_. (a) Non-treated cells; (b) vascular endothelial growth factor treated cells (20 μg/mL); (c) *Ocimum Sanctum* ethanolic extract (OSE) treated cells (50 μg/mL); (d) OSE treated cells (70 μg/mL); (e) OSE treated cells (100 μg/mL); (f) OSE treated cells (200 μg/mL); (g) cRGD treated cells (20 μg/mL). Microphotograph taken on 10×.

Angiogenesis is characterized by cell migration to designated sites of new blood vessel formation. We investigated the effect of OS on the tube formation of A549 cells. On a matrix-coated surface, A549 cells are capable of forming tubules through connecting to neighboring cells in the presence of VEGF (positive control) (Figure-1). However, no tube formation was observed following treatment with OS extract or cRGD as a negative control as well as the cells on the non-coated matrix area (non-treated cells) (Figure-1). The intercellular connection was restricted and A549 cells failed to form tubes.

### OS extract decreases expression of integrin αvβ3 in A549 cells

**Figure-2:**
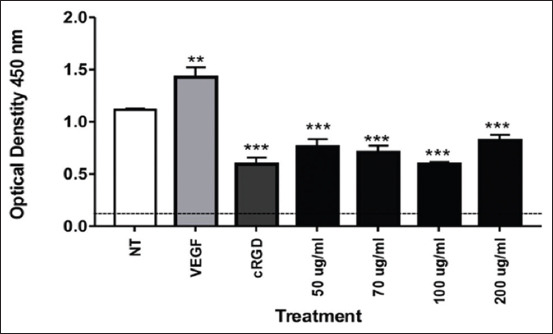
*Ocimum sanctum* ethanolic extract significantly decreases the expression of integrin αvβ3 in A549 cells. A549 cell lysates were analyzed using the human ITG αvβ3 enzyme-linked immunosorbent assay kit. Statistical analysis was performed by one-way analysis of variance followed by Tukey’s *post hoc* test as appropriate (****p<0.05=significant).

We analyzed expression of the αvβ3 integrin using ELISA to further understand the antiangiogenic effect of OS. Our results demonstrated that OS at 50 μg/mL, 70 μg/mL, and 100 μg/mL significantly suppressed αvβ3 expression in A549 cells similar to the positive control (cRGD) (p<0.05) (Figure-2). Interestingly, treatment of A549 cells with 200 μg/mL OS demonstrated a slight increase in the expression of αvβ, although it was still significantly decreased compared to the negative control NT cells (Figure-2).

### OS extract suppresses MMP-2 and MMP-9 expression in A549 cells

**Figure-3:**
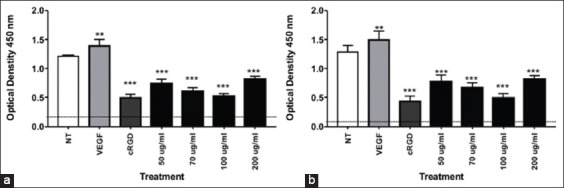
(a and b) *Ocimum sanctum* extract significantly decreases A549 expression of matrix metalloproteinase (MMP)-2 and MMP-9. A549 lysates were analyzed using human MMP-2 and MMP-9 enzyme-linked immunosorbent assay kits. Statistical analysis was performed by one-way analysis of variance followed by Tukey’s *post hoc* test as appropriate (****p<0.05=significant).

MMP-2 and MMP-9 ELISAs were used to further validate the role of OS ethanolic extract in angiogenesis of A549 cells. The results demonstrated that OS at 50 μg/mL, 70 μg/mL, and 100 μg/mL significantly depressed the expression of both MMP-2 and MMP-9, with the greatest effect observed following treatment with 100 μg/mL. The expression of MMP-2 and MMP-9 tended to slightly increase with an OS dosage of 200 μg/mL; however, the decrease was still significant compared to the negative control (NT cells) (Figure-3).

## Discussion

Angiogenesis facilitates formation of new blood vessels from pre-existing blood cells through the sprouting of endothelial cells and expansion of the vascular tree [5,18]. Although angiogenesis has important roles in normal physiology, such as during embryo development, wound healing, and collateral formation to improve organ function, angiogenesis is also associated with various disorders, including cancer [7]. Therefore, angiogenesis is considered to be a valid target for the treatment of many solid tumors and metastases [19].

**Figure-4:**
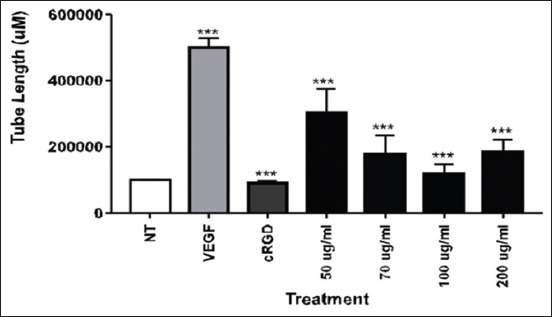
*Ocimum sanctum* (OS) ethanolic extract suppresses tube formation of human lung adenocarcinoma (A549) cells. OS ethanolic extract was give into the cells, and vascular endothelial growth factor was run as positive controls; meanwhile, cRGD was run as negative control. Tube formation was assessed by the use of a stage micrometer as calibrator. Data are given as mean±SD for n=3 independent experiments. Statistical analysis was performed using one-way analysis of variance followed by Tukey’s *post hoc* test as appropriate (****p<0.05=significant).

In this study, we observed the ability of OS ethanolic extract to prevent angiogenesis of A549 cells. Our analysis demonstrated that OS treatment at all concentrations tested led to failure of A549 cells to form tubules in an angiogenesis assay (Figures-1 and 4). To elucidate this mechanism, we also examine the expression of αvβ3 integrin, MMP-2, and MMP-9 by ELISA. We found that the expression of αvβ3 integrin, MMP-2, and MMP-9 was reduced following treatment with OS (Figures-2 and 3).

Angiogenesis is initiated following the disruption of the endothelial cell basement membrane and ECM by proteolytic enzymes [20]. The interaction between MMPs, mainly MMP-2, MMP-9, and αvβ3 on the cell surface, is important for the induction of signaling during angiogenesis. MMPs represent a highly regulated family of structurally related enzymes that are capable of degrading components of the ECM. MMP2 and MMP9, secreted by EPO-activated endothelial cells, are believed to be particularly important in angiogenesis due to their roles in cell migration and angiogenesis sprouting [21,22]. Meanwhile, the αvβ3 integrin is most abundantly expressed on angiogenic endothelial cells in remodeling and in pathological tissue. Many studies have used selective expression of αvβ3 integrin to directly target angiogenesis. Integrin binding to soluble fragments of extract may act as a decoy to inhibit physical connection and suppress the signaling events that lead to cell survival, migration, and proliferation [8]. Integrins are well-known mediators in the attachment of cells to each other as well as to the surrounding ECM [9]. In particular, αvβ3 integrin has important roles in several pathologic processes important to tumor growth and metastasis [23]. In line with our results, some studies have reported that the inhibition of αvβ3 integrin, as well as the inhibition of MMP-2 and MMP-9, decreases cellular migration and angiogenesis and may promote antiangiogenic and antitumor effects [24,25].

Silletti *et al*. [26] proposed that the use of an organic molecule may block the interaction of MMP-2 and αvβ3 and prevent tube formation. In addition, Danciu *et al*. [27] reported that increased anticancer activity was correlated with the increase in polyphenol compounds. Many studies have investigated OS ethanolic extract and demonstrated that it comprises a broad range of polyphenols and flavonoids [17,28,29]. Based on this data, we postulate that these polyphenols and flavonoids may have roles in disrupting the interaction between MMP-2, MMP-9, and αvβ3 integrin, or alternatively, may directly decrease the expression of MMP-2, MMP-9, and αvβ3 integrin.

## Conclusion

Our study demonstrates that OS ethanolic extract inhibits angiogenesis of A549 cells by reducing expression of αvβ3 integrin, MMP-2, and MMP-9. Further studies using compound isolation from the extract are required to fully validate our findings and elucidate the exact mechanisms of how OS exerts its antimetastatic activity.

## Authors’ Contributions

HW and DLK designed the experiments and study. PH and UK performed the experiments. PH, UK, and HW interpreted the data. TT, SS, DLK, and HW drafted and revised the manuscript. All authors read and approved the final manuscript.
